# Cell-specific expression of the analgesic-antitumor peptide coding sequence under the control of the human α-fetoprotein gene promoter and enhancer

**DOI:** 10.3892/etm.2015.2166

**Published:** 2015-01-02

**Authors:** SISI JIN, XIANFAN LIN, HUAQIN GUAN, JINMING WU

**Affiliations:** Department of Gastroenterology, The First Affiliated Hospital of Wenzhou Medical College, Wenzhou, Zhejiang 325000, P.R. China

**Keywords:** analgesic-antitumor peptide, α-fetoprotein, hepatocellular carcinoma, gene therapy

## Abstract

The aim of the present study was to construct a gene-modified hepatocellular carcinoma (HCC)-specific analgesic-antitumor peptide (AGAP) expression vector regulated by the α-fetoprotein (AFP) promoter and enhancer, in order to evaluate its effect. The AFP promoter is generally used in HCC-specific gene therapy strategies. However, this approach is limited by the weak activity of the AFP promoter. Linking the AFP enhancer and promoter has been shown to generate a stronger and more HCC-selective promoter. The AGAP DNA fragment was amplified from the total RNA of the Chinese scorpion, *Buthus martensii* Karsch. The fragment was subsequently cloned into the pAFP plasmid with the minimal essential DNA fragment, which included the AFP gene promoter and enhancer, to construct the recombinant plasmid, pAFP-AGAP. The plasmid was transfected into HepG2 cells and the mRNA expression levels of AGAP were detected by reverse transcription polymerase chain reaction (RT-PCR). In addition, Cell Counting Kit 8 (CCK-8) was used to analyze the cytotoxicity of plasmid transfection. The length, position and orientation of the inserted AGAP gene were all confirmed to be correct; thus, the recombinant vector was successfully constructed. Using RT-PCR and CCK-8 analysis, the mRNA expression levels of AGAP and the cytotoxicity in AFP-producing human HCC cells were determined. The AFP promoter and enhancer were found to specifically accelerate the expression of the target genes within the cells that were positive for AFP. Therefore, the method used in the present study was demonstrated to be a novel integration of traditional Chinese medicine with western medicine.

## Introduction

Hepatocellular carcinoma (HCC) is one of the most common types of malignancy, with poor prognosis in East Asian populations, particularly in China. The annual number of mortalities worldwide is estimated as 250,000. Multiple treatment modalities have been applied to HCC; however, the condition remains one of the most difficult tumors to treat when multiple foci of the tumor or distant metastases are present (1,2). Thus, novel treatment modalities are urgently required (3).

Since gene therapy clinical trials began in 1990, gene therapy modalities for malignancies have been investigated extensively (4). One approach is based on the insertion of a suicide gene. The analgesic-antitumor peptide (AGAP) gene is found in the venom of the scorpion, *Buthus martensii* Karsch. AGAP has been shown to exert analgesic and antitumor activities, indicating that the gene has potential in clinical situations as an antitumor drug (5). The α-fetoprotein (AFP) gene is normally expressed in fetal livers and is transcriptionally silent in adult livers; however, the gene is known to be overexpressed in human HCC patients (6). Thus, the AFP promoter is generally used in HCC-specific gene therapy strategies (7). However, this approach is limited by the weak activity of the AFP promoter. Linking the AFP enhancer and promoter has been shown to generate a stronger and HCC-selective promoter (8). These observations indicate that utilization of AFP promoter and enhancer driven expression in a plasmid vector can confer the selective expression of a heterologous suicide gene in HCC cells. Therefore, it may be feasible to produce a high local concentration of AGAP at the tumor site by targeting the drug in a tumor cell-specific manner, subsequently resulting in the targeted killing of cancer cells (9,10).

## Materials and methods

### Plasmid construction

Total RNA of the Chinese *Buthus martensii* Karsch scorpion was used to amplify the AGAP DNA fragment by reverse transcription polymerase chain reaction (RT-PCR). For amplification, a 100-mg sample of fresh scorpion tail was prepared, and the total RNA was isolated using TRIzol reagent, according to the manufacturer’s instructions (Takara Bio, Inc., Tokyo, Japan). The present study was approved by the Ethics Committee of the First Affiliated Hospital of Wenzhou Medical College (Wenzhou, China). The RNA was detected with a UV spectrophotometer (UV1900; Shanghai Shangtian Precision Instrument Co., Ltd., Shanghai, China). Using the full sequence of the AGAP gene obtained from GenBank (accession no. AF464898), the following primers were designed and synthesized: AGAP forward, 5′-CATGCCATGGCCGTACGCGATGGTTATATTGCCG-3′ (containing an *Nco*I site), and reverse, 5′-TGCTCTAGAGAC CGCCATTGCATTTTCCTG-3′ (containing an *Xba*I site).

The conditions for PCR (Takara Bio, Inc.) were as follows: Initial denaturation at 94°C for 5 min (one cycle), followed by 28 cycles of 94°C for 30 sec, 54°C for 30 sec and 72°C for 60 sec, and a final elongation at 72°C for 5 min. The final product was visualized by 10% agarose gel electrophoresis (Biowest, Nuaillé, France).

**Figure 1:**
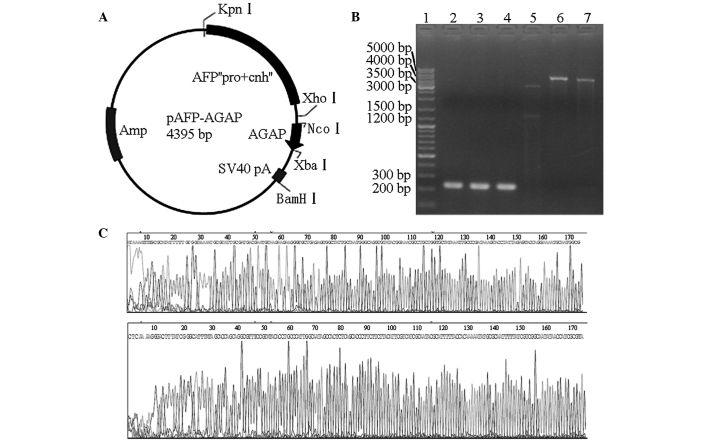
Verification of the recombinant pAFP-AGAP plasmid. (A) Construction map of the pAFP-AGAP plasmid. (B) Electropherogram of the DNA. Lanes: 1, marker; 2–4, AGAP DNA fragments; 5, pAFP-AGAP digested with *Kpn*I and *Xba*I; 6, pAFP-AGAP digested with *Xba*I; 7, pAFP-AGAP digested with *Nco*I and *Xba*I. (C) Forward and reverse sequences of the recombinant plasmid. AFP, α-fetoprotein; AGAP, analgesic-antitumor peptide.

The pAFP plasmid was provided by Dr Cheng and Dr Cristian Smerdou, and contained the minimal essential 1,002-bp DNA fragment with the AFP promoter and enhancer (11). The AGAP DNA fragment was subsequently inserted into the *Nco*I-*Xba*I site of the pAFP plasmid, producing the pAFP-AGAP plasmid (Fig. 1A). In this resulting construct, the synthesis of the AGAP protein largely depended on the human AFP promoter (217 bp) and enhancer (785 bp) to mediate transcription. The pAFP-AGAP plasmid was transformed into *Escherichia coli* DH5α cells, and then screened and verified by PCR and restriction enzyme digestion (Thermo Fisher Scientific, Vilnius, Lithuania). The sequence of the AGAP gene in the pAFP-AGAP plasmid was confirmed at Takara Bio, Inc.

### Cell culture

HepG2 (Bogoo, Inc., Shanghai, China) is a human AFP-producing hepatoma cell line. The cells were maintained in Dulbecco’s modified Eagle’s medium (DMEM; HyClone; GE Healthcare Life Sciences, Logan, UT, USA) that was supplemented with 10% fetal bovine serum (FBS; HyClone).

### Transfection and mRNA expression of AGAP

At one day prior to transfection, 2×10^5^ cells were plated in 2 ml growth medium without antibiotics and seeded into six-well plates (Costar^®^; Corning Life Sciences, Cambridge, MA, USA). After overnight cultivation, the cells were divided into two groups: pAFP-AGAP group and pAFP group. The medium was then removed and the cells were transfected with a DNA-lipid complex. The complex consisted of a 250-μl solution containing 4 μg pAFP-AGAP (or pAFP), a 250-μl Lipofectamine^®^ 2000 (Invitrogen Life Technologies, Carlsbad, CA, USA) solution containing 10 μl stock solution (1:1), and 2 ml DMEM without FBS. The cells were incubated with the DNA-lipid complex at 37°C for 6 h, washed and supplemented with 2 ml complete medium containing 10% FBS. Cells were harvested at 24, 48 and 72 h after cultivation in an incubator with 95% humidified air and 5% CO_2_ at 37°C.

Total RNA was isolated from the cells using RNAiso Reagent (Takara Bio, Inc.), according to the manufacturer’s instructions. The mRNA expression of AGAP was analyzed by RT-PCR using the forward and reverse primers of AGAP, as described previously. The final product was visualized by 10% agarose gel electrophoresis.

### Cytotoxicity assay

HepG2 cells were maintained and transfected with the plasmids using Lipofectamine^®^ 2000 as follows. A 100-μl cell suspension was dispensed in a 96-well plate (5,000 cells/well). Following overnight cultivation, the medium was removed and the cells were divided into two groups: pAFP-AGAP group and pAFP group. The cells were then transfected with a DNA-lipid complex. The DNA-lipid complex was freshly obtained by adding a 25-μl solution of 0.2 μg pAFP-AGAP (or pAFP) to 25 μl Lipofectamine^®^ 2000 solution, containing 0.5 μl stock solution (1:1). The cells from both groups were then diluted in 100 μl DMEM without FBS. The cells were incubated with the DNA complex at 37°C for 6 h, washed and supplemented with 100 μl complete medium containing 10% FBS. The plates were incubated for 24, 48 or 72 h in 95% humidified air with 5% CO_2_ at 37°C.

Cell Counting Kit 8 (CCK-8) solution (10 μl; Dojindo, Kyushu, Japan) was added to each well of the plate and the cells were further cultured for 1 h at 37°C. The color reaction was quantitated using an automatic plate reader (ELx800G; DIALAB GmbH, Inc., Vienna, Austria) at 450 nm with a reference filter of 630 nm. The results were expressed as the mean value of three wells. The percentage of growth inhibition was calculated as follows: Growth inhibition = (A − B)/(A − C) × 100%, where A is the absorbance from the cells incubated with the medium containing pAFP, B is the absorbance from the cells incubated with the medium containing pAFP-AGAP and C is the absorbance from the cells incubated with medium alone.

### Statistical analysis

Results are expressed as the mean ± standard deviation. Statistical analyses were conducted using SPSS version 12.0 software (SPSS Inc., Chicago, IL, USA). Data from all the experiments were statistically analyzed using the Student’s t-test, where P<0.05 was considered to indicate a statistically significant difference.

## Results

### Verification of the recombinant eukaryotic expression vector

Through RT-PCR analysis of the scorpion genetic material, the AGAP gene sequence was determined to be ~220 bp. The final products of the AGAP DNA fragments following RT-PCR were consistent with the expected length, as shown by electrophoresis (Fig. 1B). Thus, the AGAP gene sequence was cloned into the pAFP plasmid to obtain the eukaryotic expression vector, pAFP-AGAP (Fig. 1A). Subsequently, the pAFP-AGAP plasmid was digested with *Xba*I to produce a 4,395-bp fragment (lane 6). The plasmid was digested with *Kpn*I and *Xba*I to produce 3,118 bp and 1,277 bp fragments (lane 5). The plasmid was also digested with *Nco*I and *Xba*I to produce 220 bp and 4,175 bp fragments (lane 7). Each fragment was detected on the electropherogram, and indicated that the construction of the pAFP-AGAP vector was correct by multi-enzyme digestion (Fig. 1B). In addition, the sequence of the AGAP gene in the pAFP-AGAP plasmid was confirmed (Fig. 1C). The forward and reverse sequences of the recombinant plasmid exhibited 98% similarity with the GenBank sequences. The difference may have been caused by gene mutation or the mismatch of gene cloning.

### Transfection and mRNA expression of AGAP

**Figure 2:**
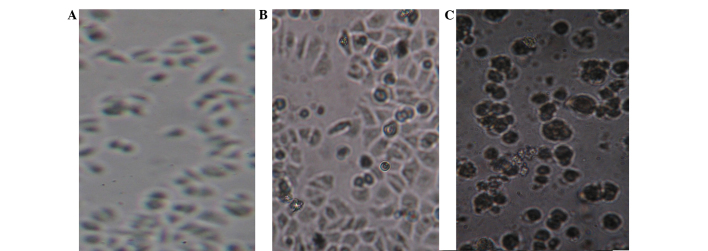
HepG2 cells were transfected with the pAFP-AGAP plasmid and harvested at (A) 24, (B) 48 and (C) 72 h. AFP, α-fetoprotein; AGAP, analgesic-antitumor peptide.

**Figure 3:**
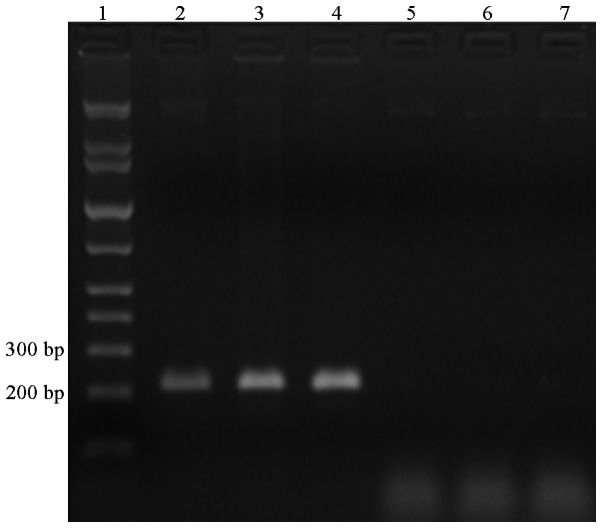
mRNA expression of AGAP in the HepG2 cells. Lanes: 1, marker; 2–4, HepG2 cells were transfected with pAFP-AGAP at 24, 48 and 72 h, respectively; 5–7, HepG2 cells were transfected with pAFP at 24, 48 and 72 h, respectively. AFP, α-fetoprotein; AGAP, analgesic-antitumor peptide.

HepG2 cells were transfected with the pAFP-AGAP complex. The cells grew normally after 24 h (Fig. 2A); however, cellular swelling and cell suspension was observed 48 h after cultivation (Fig. 2B). The presence of floating cells, cytoplasmic granulations and vacuolation was observed after 72 h (Fig. 2C). This phenomenon indicated that the cytotoxicity increased gradually over time. The AGAP gene was amplified using RT-PCR from the RNA of the cells simultaneously. The products following RT-PCR of the HepG2 cells transfected with the pAFP-AGAP plasmid for 24, 48 and 72 h were confirmed to be the correct size by gel electrophoresis. However, the same product was not found on the electrophoresis gel following RT-PCR of the HepG2 cells transfected with the pAFP plasmid only (control; Fig. 3).

### CCK-8 analysis

Cytotoxicity was detected in the HepG2 cells following transfection with the pAFP-AGAP plasmid or the pAFP complex for 24, 48 and 72 h. The percentage of growth inhibition was found to be 2.4, 44.4 and 74.6% at 24, 48 and 72 h, respectively. Thus, growth inhibition was shown to increase gradually over time (Table I).

## Discussion

Scorpions have been used in traditional Chinese medicine for a number of years (12), with scorpion venom applied for the treatment of convulsions and epilepsy since the Sung Dynasty (960-1279 A.D.) (13). AGAP has been purified from the venom of *Buthus martensii* Karsch, which is a widely distributed scorpion species in China. Previous studies have demonstrated that AGAP exhibits analgesic and antitumor activities (14,15). Gu *et al* (14) demonstrated that AGAP could inhibit colon cancer cell growth. In addition, AGAP has been shown to prolong the survival times of mice that have undergone Ehrlich ascites tumor cell engraftment, whilst effectively inhibiting S-180 fibrosarcoma growth (5). Therefore, the present study investigated AGAP as a suicide gene for use in gene therapy.

The AFP gene encodes the major serum protein in the developing mammalian fetus, with AFP gene expression observed in the visceral yolk sac endoderm, the fetal liver, and to a much lower degree, in the fetal gut and kidney (16). Under physiological conditions, the AFP gene is expressed at extremely low levels in the adult liver; however, gene expression can be reactivated during periods of renewed cell growth, including during liver regeneration and in HCC (6). A previous study demonstrated that AFP expression is frequently upregulated in liver cancer cells (17). In addition, Marrero *et al* (18) showed that diptherotoxin inhibited hepatocellular carcinoma under the control of the AFP promoter; however, this approach was limited by the weak activity of the AFP promoter. It is known that effective control of downstream genes is dependent on the promoter and enhancer working together. Thus, the present study utilized the AFP promoter and enhancer to construct a gene-modified HCC-specific AGAP expression vector.

In conclusion, the results of the present study indicated that the AGAP gene was successfully integrated into the pAFP plasmid and expressed. The AGAP gene was specifically expressed at very high levels in human HCC tumor cells. Therefore, there is a potential industrial application of inducing AGAP expression through eukaryotic expression vectors. Similarly to the use of the AFP promoter and enhancer for HCC, prostate-specific antigen promoters have been used for prostate cancer (19), while E2F and telomerase reverse transcriptase promoters are used for various types of tumors (20,21). These additional promoters may be used in place of AFP to adapt this novel strategy to different types of tumors.

However, future studies are required and should focus on the detection of expression at the protein level, in addition to *in vivo* analysis.

## Figures and Tables

**Table I:** CCK-8 analysis showing the OD values of the HepG2 cells transfected with the various plasmids.

Parameter	24 h	48 h	72 h
Transfection with pAFP	0.42±0.05	0.81±0.07	1.50±0.13
Transfection with pAFP-AGAP	0.41±0.04	0.45±0.02	0.38±0.04
P-value	0.97	<0.001	<0.001

Results are expressed as the mean ± standard deviation. CCK-8, Cell Counting Kit 8; OD, optical density; AFP, α-fetoprotein; AGAP, analgesic-antitumor peptide.
